# Positive Regulation of DNA Double Strand Break Repair Activity during Differentiation of Long Life Span Cells: The Example of Adipogenesis

**DOI:** 10.1371/journal.pone.0003345

**Published:** 2008-10-10

**Authors:** Aline Meulle, Bernard Salles, Danièle Daviaud, Philippe Valet, Catherine Muller

**Affiliations:** 1 Université de Toulouse, Toulouse, France; 2 Institute of Pharmacology and Structural Biology CNRS UMR 5089, Toulouse, France; 3 Institut National de la Santé et de la Recherche médicale, INSERM U858 Equipe 3, IFR31, Toulouse, France; Ordway Research Institute, United States of America

## Abstract

Little information is available on the ability of terminally differentiated cells to efficiently repair DNA double strand breaks (DSBs), and one might reasonably speculate that efficient DNA repair of these threatening DNA lesions, is needed in cells of long life span with no or limited regeneration from precursor. Few tissues are available besides neurons that allow the study of DNA DSBs repair activity in very long-lived cells. Adipocytes represent a suitable model since it is generally admitted that there is a very slow turnover of adipocytes in adult. Using both Pulse Field Gel Electrophoresis (PFGE) and the disappearance of the phosphorylated form of the histone variant H2AX, we demonstrated that the ability to repair DSBs is increased during adipocyte differentiation using the murine pre-adipocyte cell line, 3T3F442A. In mammalian cells, DSBs are mainly repaired by the non-homologous end-joining pathway (NHEJ) that relies on the DNA dependent protein kinase (DNA-PK) activity. During the first 24 h following the commitment into adipogenesis, we show an increase in the expression and activity of the catalytic sub-unit of the DNA-PK complex, DNA-PKcs. The increased in DNA DSBs repair activity observed in adipocytes was due to the increase in DNA-PK activity as shown by the use of DNA-PK inhibitor or sub-clones of 3T3F442A deficient in DNA-PKcs using long term RNA interference. Interestingly, the up-regulation of DNA-PK does not regulate the differentiation program itself. Finally, similar positive regulation of DNA-PKcs expression and activity was observed during differentiation of primary culture of pre-adipocytes isolated from human sub-cutaneous adipose tissue.

Our results show that DNA DSBs repair activity is up regulated during the early commitment into adipogenesis due to an up-regulation of DNA-PK expression and activity. In opposition to the general view that DNA DSBs repair is decreased during differentiation, our results demonstrate that an up-regulation of this process might be observed in post-mitotic long-lived cells.

## Introduction

The integrity of DNA is constantly being challenged by exogenous DNA-damaging agents but also by endogenously produced radicals, stalled replication forks and by spontaneous formation of abasic sites in DNA. Mammalian cells have evolved a number of repair systems to deal with these various types of DNA damage. Schematically, damage to individual bases and single strand breaks can be repair by base excision repair (BER), bulky adducts and photoproducts by nucleotide excision repair (NER) and DNA double strand breaks can be repaired by homologous recombination (HR) or non homologous end joining (NHEJ) [1], [2]. Defects in one of these DNA repair mechanisms can therefore result in increased cell death and genomic instability leading to disease such as cancer [3].

While most of the studies have investigated the response of proliferating cells to genotoxic agents, less information exists on the DNA repair activity in differentiated cells that are highly represented in multicellular organisms. Differentiated cells are still transcriptionnally active and there is a need to maintain genome integrity of the transcribed genome through the life span. In addition, DNA repair activities are needed to protect cells from death and to therefore ensure tissue homeostasis. It has been previously described that differentiated cells use specific strategies such as increase efficiency of the repair of active genes to ensure the maintenance of the transcriptionnally active domains in the presence of bulky adducts and photoproducts [4], [5]. However, not all DNA repair systems are coupled to transcription, and to date NER (the pathway involved in the reparation of these lesions) is the only pathway for which transcription coupled repair has been formally demonstrated.

Of the various type of damage that arise within the cell, DNA double strand (DSBs) are considered as the most lethal form of DNA damage, which, if left unrepaired will lead to cell death [6], [7]. DSBs are generated by environmental factors such as ionizing radiation, by cellular metabolic products and as recombination intermediates [3]. Mammalian cells have evolved two main mechanisms for the repair of DSBs: homologous recombination (HR) and non-homologous end joining (NHEJ) [7]–[10]. During HR, the chromosome carrying the DSBs must enter into synapses with an undamaged molecule with which it shares extensive homology and copies the nucleotide sequence around the break. The entire process is slow and is thought to occur preferentially during the late S and G2 phases of the cell cycle, where the availability of a sister chromatid facilitates repair [10]. By contrast, NHEJ, the predominant pathway in mammalian cells, which simply pieces together the broken DNA ends, has no such requirement and can therefore be operational in all phases of the cell cycle [11]. This form of repair is extremely efficient and requires a kinase specifically activated by DNA ends, the DNA dependent protein kinase (DNA-PK) [12]. The DNA-PK holoenzyme comprises the Ku heterodimer, which binds to DNA double strand breaks, recruiting and activating the catalytic sub-unit, DNA-PKcs [13]. DNA-PK together with the XRCC4/DNA ligase IV/XLF complex and the recently identified co-factors Artemis is specifically required for the repair of DNA double-strand breaks by the NHEJ pathway [6], [7]. Accordingly, numerous studies have shown that cells lacking either functional DNA-PKcs or Ku through mutations or gene knock-out are hypersensitive to IR due to a defect in DNA double strand breaks repair [6], [7]. Little information is available on the ability of terminally differentiated cells to efficiently repair DNA DSBs, and one might reasonably speculate that efficient DNA repair is needed in cells of long life span with no or limited regeneration from precursor. Limited studies have been performed in mature post-mitotic neurons and there is evidence that DSBs repair still occurred in these cells [14], [15] although the process is slower than in astrocytes [16]. Few tissues are available, besides neurons that allow the study of DNA DSBs repair activity in very long-lived cells. Thus, adipocytes represent a suitable model since it is generally admitted that there is a very slow turnover of adipocytes in adult [17]. In the absence of weight gain, they might turn over within months or years or might in fact not turn over at all [18]. To address the question of the potential differentiation-associated regulation of DNA DSBs repair activity, we took advantage of the well-established murine 3T3F442A preadipocyte cell line, which has been largely validated as a valuable model of adipogenesis [19]. Our results show that adipocyte differentiation is associated with an increase in NHEJ activity highlighting for the first time a clear association between the commitment into differentiation of long lived cells and up-regulation of DNA DSBs repair activity.

## Materials and Methods

### Cells, cell culture and induction of differentiation

The murine 3T3F442A pre-adipocyte cell line was cultured in DMEM (Invitrogen, Auckland, NZ) supplemented with 10% fetal calf serum (FCS), 2 mM glutamine and antibiotics. The differentiation was induced by incubating confluent cells in differentiation medium (DMEM supplemented with 10% FCS plus 50 nM insulin) as described previously for up to 14 days [20]. Unless indicated, the term “adipocytes” represent cells that have been differentiated for 7 to 10 days. The murine Balb-C cell line was cultured in αMEM medium (Invitrogen, Auckland, NZ) supplemented with 10% FCS, 2 mM glutamine and antibiotics.

The human preadipocytes were isolated from human subcutaneous adipose tissue obtained from patients undergoing abdominal lipectomy at the plastic surgery department of Rangueil Hospital (Toulouse, France) under the agreement of local ethic committee. Adipose tissue pieces were immediately used for collagenase digestion as previously described [20]. The digestate was centrifuged to separate adipocytes from the stroma-vascular fraction, containing pre-adipocytes (pellet). Cells isolated from the SVF fraction were induced to differentiate into adipocytes as previously described [21]. Briefly, the stromal vascular pellets were incubated overnight with DMEM/Ham's F12 medium supplemented with 10% FCS. After overnight culture, the medium was replaced by a serum-free adipogenic medium (DMEM/Ham's F12 (1∶1) medium supplemented with 10 mg/ml transferrin, 33 mM biotin, 66 mM insulin, 1 nM triiodothyronine, and 17 mM pantothenate) and that time point was considered as day 0. In indicated experiment, perigonadic adipose tissue of Balb-C and SCID mice were removed and freshly isolated tissues were fixed with phosphate-buffered formalin overnight, then paraffin wax embedded and subsequently deparaffinized. Sections of 5 µm were obtained and counterstained with hematoxylin (Sigma-Aldrich, Saint-Louis, USA).

To obtain pre-adipocyte clones with long term down regulation of DNA-PKcs expression, the 3T3F442A cells were transfected using Amaxa electropation system (Nucleofector solution V; program: T030) with EBV-based vectors containing either control or short hairpin RNA coding sequences for murine DNA-PKcs as previously described [22]. Transfected cells were incubated, at low density, in the presence of hygromycin (600 µg/ml) and 2 individual clones with highly significant reduction of DNA-PKcs expression were obtained (further named DNA-PKcs^KD^ in our study, KD standing for kinase dead). To induce the differentiation, the 3T3F442A control or DNA-PKcs^KD^ clones obtained were grown to confluence and incubated in a differentiation medium (DMEM supplemented with 10% FCS, plus 50 nM insulin, plus 30 nM rosiglitazone). In indicated experiments, the differentiation was induced in the presence of the DNA-PKcs inhibitor NU7026 (Calbiochem, Darmstadt, Germany) at 20 µM final.

### Pulsed-field gel electrophoresis

Preadipocytes and adipocytes cells were incubated with 500 pM of calicheamicin γ1 (CLγ1, a generous gift from Dr. P.R. Hamann, Wyeth Research, Pearl River, NY) during 1 h in serum-free medium. After 1 h, complete medium was added and cells were post-incubated at indicated times. In certain experiments preadipocytes cells and adipocytes were irradiated using a ^137^Cs source irradiator (4.4 Gy/min, Biobeam 8000) and cells were post-incubated following irradiation at different time between 0.5 and 24 h. At chosen times for the PFGE analysis, cells were rinsed with PBS, trypsinized, centrifuged and resuspended in PBS containing low-melting-point agarose (Bio-Rad, Hercules, CA) to obtain a 0.5% final concentration. After solidifying in 3-mm plastic tubing at 4°C, the plugs were further processed at 4°C to minimize the occurrence of heat-induced strand breaks [23]. The first incubation was performed in lysis buffer (0.5 M EDTA, 2% N-lauroylsarcosine, 1 mg/mL proteinase K per ml) at 4°C for 20 to 24 h. Following protein lysis, the plugs were incubated for an additional 20 to 24 h in Buffer HS (1.85 M NaCl, 0.15 M KCl, 5 mM MgCl_2_, 2 mM EDTA, 4 mM Tris-HCl pH 7.5, 0.5% triton-X 100, pH 7.5), and washed three times with 0.1 M EDTA pH 8.0. After this treatment, the plugs were loaded onto a 0.8% agarose gel (Bio-Rad, Hercules, CA), and electrophoresed for 30 h with a pulse linear program (200 s at 1800 s, 4 V/cm, 11 °C, angle 120°, TBE 0.5×). Gels were stained with SYBER-Green (Moleculars Probes, Eugene, OR), and the fluorescence detected and analyzed on a Typhoon fluorimager (Molecular Dynamics). The percentage of DNA released was determined as described [24]. In indicated experiments, preadipocytes and adipocytes cells were incubated with 50 µM of wortmannin (Sigma-Aldrich, Saint-Louis, USA) 0.5 h before treatment with CLγ1. After extensive washing, cells were post-incubated for the indicated times in complete medium containing wortmannin.

### Cell extracts, Western blotting and immunofluorescence

Whole-cells extract were prepared as previously described [25]. Briefly, cell pellets were resuspended in extraction buffer (50 mM NaF, 20 mM HEPES pH 8, 450 mM NaCl, 25% v/v glycerol, 0.2 mM EDTA, 0.5 mM DTT, 0.5 mM PMSF, 0.5 µg/ml leupeptin, 0.5 g/ml protease inhibitor and 1.0 µg/ml trypsin inhibitor), subjected to three freeze/thaw cycles (liquid nitrogen/37°C), and centrifuged at 1000 rpm for 10 min at 4°C. Supernatants were stored at −80°C prior to use and protein concentrations were determined by Bradford's method. Western blots were performed as previously described [25]. Detection of γH2AX foci by immunofluorescence to observe H2AX foci, was performed as previously described [26]. Fluorescence images were captured by using a Nikon eclipse TE 300 fluorescence microscope with excitation at 488 nm for FITC (H2AX) and 405 nm for DAPI (100× oil immersion objective), equipped with a Nikon digital camera DXM 1200. Foci were counted by eye in 100 cells per time point and results were obtained from three independent experiments for pre-adipocytes and adipocytes cells. The following antibodies were used: polyclonal anti-H2A and monoclonal anti-H2AX antibody (clone JBW 301) both obtained from Upstate Biotechnology (Charlottesville, VA, USA), polyclonal anti-XRCC4 antibody (Serotec Ltd. Oxford, UK), polyclonal anti-HSL antibody (Santa Cruz Biotechnologies, CA, USA), polyclonal anti-Rad-51 (a kind gift from Dr M. Defais, IPBS, Toulouse, France), polyclonal anti-Cernunos/KLF (Bethyl Laboratories), polyclonal anti-Artémis (a kind gift of Dc Steve M. Yannone, LBL, Berkeley, USA), monoclonal anti-ATM antibody (clone 2C1, Genetex, Zeeland, MI), anti-Ku70 (clone N3H10), anti-Ku80 (clone S10B1), anti-DNA-PKcs (clone 18-2), anti-PAN actin (clone ACTN05), anti-tubulin (clone DM1A) monoclonal antibodies all obtained from Neomarkers (Interchim, Montluçon, France).

### DNA-PK assay

Kinase assays were performed as previously described [27]. Briefly, after incubation of the 250 µg of protein extracts with double-stranded DNA-cellulose beads (Sigma-Aldrich, Saint-Louis, USA), beads were extensively washed with Z' 0.05 buffer [25 mM HEPES (pH 7.9), 50 mM KCl, 10 mM, MgCl2, 20% glycerol, 0.1% Nodinet P-40, 1 mM dithio-threitol] and incubated for 1 h, in the presence or not of a wild-type peptide (EPPLSQEAFADLLKK) that is a substrate for DNA-PK. Results were expressed in fold induction compared to the DNA-PK kinase activity of exponentially pre-adipocyte extract.

### Electrophoretic mobility shift assays (EMSA)

EMSA was performed as previously described [25]. Briefly, 2 µg of pre-adipocytes or adipocytes WCEs were incubated with [γ-32P]ATP-labeleded linear probes (4 ng, 100 000 cpm) and closed circular plasmid (0.75 ug) as a competitor in 20 µl of reaction buffer (20 mM Tris-HCl pH 7.5, 2 mM MgCl2, 0.1 mM EDTA, 0.25 mM DTT, 5% glycerol). The samples underwent electrophoresis on a 5% polyacrylamide non denaturating gel. Gels were dried on Whatman 3 mm paper and autoradiographed.

### RNA extraction and quantitative RT-PCR (qPCR)

Total RNAs were extracted using the RNeasy mini kit (Qiagen GmbH, Hilden, Germany). Gene expression was analyzed using real time PCR as described previously [20]. Total RNAs (1 µg) were reverse-transcribed for 60 min at 37°C using Superscript II reverse transcriptase (Invitrogen, Auckland, NZ) in the presence of a random hexamer. A minus reverse transcriptase reaction was performed in parallel to ensure the absence of genomic DNA contamination. Real time PCR was performed starting with 25 ng of cDNA and a 300 nM concentration of both sense and antisense primers in a final volume of 25 µl using the SYBR Green TaqMan Universal PCR master mix (Applied Biosystems, Foster City, CA). Fluorescence was monitored and analyzed in a GeneAmp 7300 detection system instrument (Applied Biosystems, Foster City, CA). Analysis of 18S ribosomal RNA was performed in parallel using the ribosomal RNA control TaqMan assay kit (Applied Biosystem), or HPRT RNA (100 nM) or GAPDH RNA (100 nM) to normalize for gene expression. Oligonucleotide primers were designed using the Primer Express software (PerkinElmer Life Sciences) as previously described [20].

## Results

### Enhanced capacity to repair DNA DSBs is observed during adipocyte differentiation

**Figure 1:**
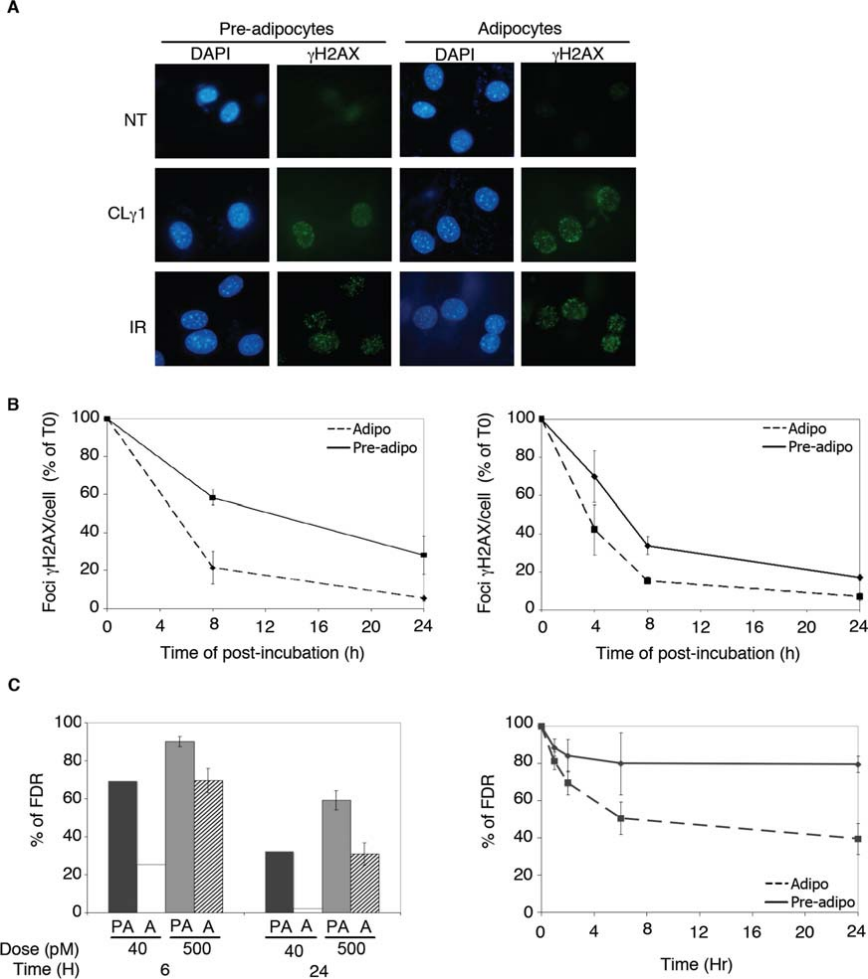
Adipocytes display an increased ability to repair DNA double strand breaks as compared to pre-adipocytes. (A) γH2AX foci formation detected by immunofluorescence using an antibody that specifically recognizes the phosphorylated form of H2AX, in pre-adipocytes and adipocytes cells, exposed for 1 h to 2 pM of CL1γ or 30 min after irradiation at 2 Gy. (B) Kinetics of repair of DNA double-strand breaks as determined by disappearance of γ-H2AX foci after irradiation at 2 Gy (left panel) or after treatment at 2 pM of CL1γ during 1 h (right panel), mean of 3 independent experiments±SEM. (C) Kinetics of DNA DSBs repair determined by pulse-field gel electrophoresis after treatment with CL1γ (40 or 500 pM, 1 h) (left panel) or after γ-irradiation at 80 Gy (right panel). Results were obtained from the quantification of two independent experiments for treatment at 40 pM and three independent experiments for 500 pM and 80 Gy (mean±SEM).

To investigate the DNA DSB repair activity of post-mitotic adipocytes, the murine pre-adipocyte 3T3F442A cell line was used. When post-confluent 3T3F442A cells are incubated in the presence of insulin and FCS, the cells progressively acquire a differentiated phenotype. Typically, by day 7, 80% of the cells have accumulated lipid droplets characteristics of adipose conversion [28]. Cells were exposed either to the radiomimetic drug calicheamicin γ1(CLγ1) or to ionizing radiation (IR) to create DSBs. Cell exposure to CLγ1, a natural hydrophobic enediyne antibiotic, has been shown to produce DSBs with selectivity and efficiency yielding a 1∶3 ratio of DNA DSB to SSBs in vivo, compared to a 1∶20 ratio for IR [26]. The number of DSBs occurring after treatment in either pre-adipocytes or differentiated adipocytes was first measured, using the phosphorylation of H2AX, a variant form of histone H2A [29]. The local phosphorylation of H2AX (γH2AX) by ATM/ATR/DNA-PK protein kinases is an early event occurring after formation of DSB and can extend megabases away from the break sites [30]. It has been initially proposed that the number of DSB correlates to that of γH2AX foci within a 1∶1 ratio [29]–[31] and that the foci number could be used as an indirect marker of DSBs occurrence. As shown in Fig. 1A, the number of γH2AX foci occurring in cells exposed for 1 h to 2 pM of CL1γ or 30 min after irradiation at 2 Gy was similar in both cell population suggesting that the initial number of DNA lesions is similar in treated undifferentiated and differentiated cells (mean foci per cell: 27.3±3.2 for pre-adipocytes and 25.2±7.3 for adipocytes in CL1γ-treated cells and mean foci per cell: 14.3±1.5 for pre-adipocytes and 12±2.1 for adipocytes in IR treated cells). This number of γH2AX foci after 2 Gy exposure is somewhat less than the 30 to 60 initial DSBs per Gy per cell (depending of the cell cycle position of the cell) expected from previous determination using biochemical methods [32]. Such a discrepancy may depend on the cell type used since the rate of radiation-induced γH2AX formation has been previously reported to be cell type dependent [33]. In addition, due to the limit of the optical microscopy, clusters of DSBs are difficult to resolve and might therefore appear as a single focus leading to an underestimation of the number of breaks [30], [34]. Similar levels of γH2AX expression were also observed between pre-adipocytes and adipocytes after exposure to IR (10 and 50 Gy) as measured by Western Blot experiments (data not shown). We have previously demonstrated that at low levels of DNA damage the loss of γH2AX was a marker of DNA DSBs repair after either IR or CLγ1 exposure [26]. As shown in Fig. 1B, the kinetic of loss of γH2AX immunoreactivity using foci numbering was significantly slower in pre-adipocytes that in adipocytes after either exposure to IR (2 Gy) (left panel) or CLγ1 (2 pM) (right panel). For example, the percent of foci remaining in CLγ1 treated cells (2 pM, 1 h) was 33±4.6 in pre-adipocytes compared to 15±1.9% in adipocytes (p<0.05) and 58±3.3 in pre-adipocytes compared to 21±8.6% in adipocytes (p<0.05) in IR-treated cells. These results suggest that the efficiency in DNA DSBs repair is increased in adipocytes as compared to pre-adipocytes. However, although detection of radiation induced foci is a useful tool to study DNA breaks formation and repair especially at low doses of IR, recent accumulating evidence suggest that γH2AX foci might also be induced by other DNA lesions than DSBs such as stalled replication forks [35] and even by stable association of single repair factors with chromatin in the absence of DNA damage [36]. In addition, it has been demonstrated that γH2AX foci do not correspond to the physical deposition of energy by high linear energy radiation [34]. Therefore, to confirm whether or not the DNA DSBs repair activity was increase in adipocytes versus pre-adipocytes, pulse field gel electrophoresis (PFGE) was used. Cells were treated either with low (40 pM) or high (500 pM) doses of CLγ1 for 1 h and post-incubated in drug-free medium for 6 to 24 h. From these experiments, we observed a significant slower kinetics of DSBs removal in pre-adipocytes as compared to adipocytes (see Fig. 1C, left panel). At 24 h, a significant higher number of residual DNA breaks was observed in pre-adipocytes as compared to adipocytes when 500 pM of CLγ1 was used (59±2.6 versus 31±6.3% respectively, p<0.05) and similar results were obtained when DNA DSBs activity was quantified in cells exposed to lower doses of CLγ1 (40 pM) (32 versus 2% respectively, mean of two experiments). Similar results were obtained in IR-treated cells (residual DNA breaks 24 h after exposure to IR: 79.4±4.4 in pre-adipocytes versus 39.5±8.1% in adipocytes, p<0.05, see Fig. 1C, right panel). In all these experiments, we assessed that the % of FDR at T0 was similar between pre-adipocytes and adipocytes (data not shown). Thus, using two different technical approaches our results show a faster kinetic of DSBs repair in adipocytes than in pre-adipocytes.

### Adipocyte differentiation is associated with an increase in DNA-PKcs expression and activity that contributes to the increased in NHEJ activity

**Figure 2:**
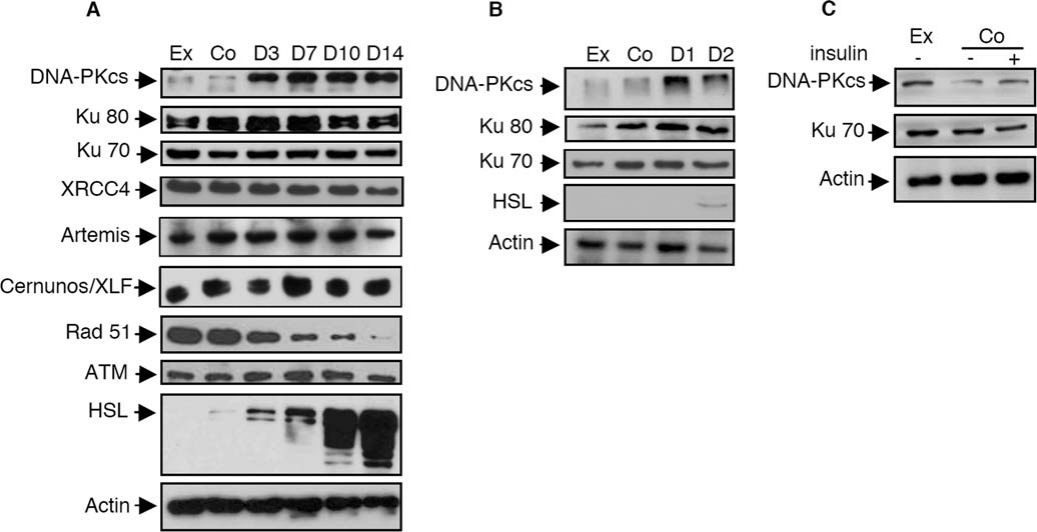
Adipocyte differentiation is associated with an early increase in DNA-PKcs expression. (A) Expression of DNA repair proteins (DNA-PKcs, Ku70, Ku80, XRCC4, Rad51 and ATM) or adipocyte differentiation marker (HSL) was analyzed by Western Blots in exponentially growing 3T3F442A fibroblast cells (Ex), in 3T3F442A fibroblast cells grown to confluence (Co) or after the indicated time of culture in adipogenic differentiation medium (B) Similar experiments were performed in exponentially growing pre-adipocytes (Ex), in pre-adipocytes grown to confluence (Co) or during the first two days of culture in adipogenic medium) (C) Expression of DNA-PKcs protein in a murine fibroblast cell line (Balb-C) grown to confluence in the presence or not of insulin in comparison to exponentially growing cells.

According to these results, we then investigated the cellular levels of the core proteins involved in the NHEJ pathway. WCEs were prepared at different time points before and after the commitment into differentiation process induced by confluence (D0). To assess our experimental conditions, we used a marker of differentiation, the hormone-sensitive lipase (HSL), which progressively increases during acquisition of an adipocyte phenotype as expected (Fig. 2A). As shown in Fig. 2A, a clear up-regulation of DNA-PKcs expression was observed between D0 and D3 following confluence whereas the levels of the other core proteins of the NHEJ process were unaffected (XRCC4, Ku70, Ku80, Cernunos/XLF, Artemis). By opposition to DNA-PKcs, the differentiation process was also without effect on the expression of another member of the Phosphatidyl-Inositol-3 kinase like kinase (PIKK) family, involved in DSBs repair and signaling [37], the Ataxia Telangiectasia Mutated protein (ATM). As stated before, DSBs are also repaired by HR, although this process is down regulated in G1 in mammalian cells [11]. We observed a strong and progressive down-regulation of one of the key factor of HR, Rad51, starting at D3 (Fig. 2A). Since the up-regulation of DNA-PKcs was observed during the first three days of the differentiation process, we investigated the occurrence of this event during this period of time. As shown in Fig. 2B, the up-regulation of DNA-PKcs occurs within the first 24 h following the commitment into the differentiation program. To further assess that neither confluence nor insulin treatment were non specifically involved in the observed effect, we use a control cell murine fibroblast cell line, BalbC. As shown in Fig. 2C, the levels of DNA-PK was unaffected by these two culture conditions.

**Figure 3:**
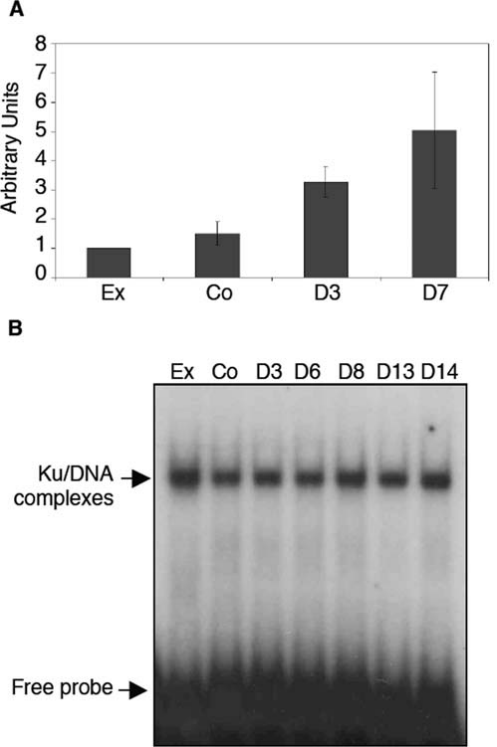
Adipocyte differentiation is associated with an increase of the kinase activity of the DNA-PK complex without modification of the activity of its DNA binding sub-unit Ku. (A) Two-hundred µg of the cellular extracts were used for the measurement of DNA-PK kinase activity, as described in the Material and Methods Section, in the pre-adipocyte cells (exponentially growing, Ex or at confluence, Co) or during adipocyte differentiation (after 3 and 7 days of culture in insulin containing medium) (mean of 5 independent experiments±SEM). (B) Ku DNA end binding activity was measured by EMSA. 10 µg of extracts obtained from the indicated cells were incubated with a [^32^P] 25-bp double-stranded DNA probe in the presence of an excess of closed circular plasmid DNA used as a non-specific competitor. The proteins/DNA complexes were resolved on a non-denaturating polyacrylamide gels and the position of the free probe and the Ku/DNA complexes are indicated.

To evaluate if the increase in DNA-PKcs expression was associated with an increase in DNA-PK activity, a specific kinase assay was performed. As shown in Fig. 3A, DNA-PK activity was significantly increased by 3.7-fold (p<0.001) in extracts from adipocytes (D3) compared with extracts from the pre-adipocyte cells (either exponentially growing or at confluence) and this difference was further maintained at latter stage of differentiation (D7). The activity of the regulatory sub-unit of DNA-PK, the Ku heterodimer was also investigated. Indeed, Ku DNA binding represents the predominant if only mechanism for DNA-PK activation because Ku-defective mutants cells have undetectable DNA-PK activity [38]. Ku DNA ends binding activity can be detected easily by using double-stranded DNA fragments in an electrophoretic mobility shift assay (EMSA) as previously described [27], [39]. As shown in Fig. 3B, no differences in Ku DNA ends binding activity were observed during adipocyte differentiation. The lack of difference in Ku activity was also found with various amounts of protein extracts (data not shown). Thus, the fact that the expression and activity of DNA-PK regulatory sub-unit Ku is unchanged during adipocyte differentiation show that the observed increase in DNA-PK activity relies on the sole increase in DNA-PKcs expression.

### The increase in DNA-PK activity explains the increased ability of adipocytes to repair DSBs

**Figure 4:**
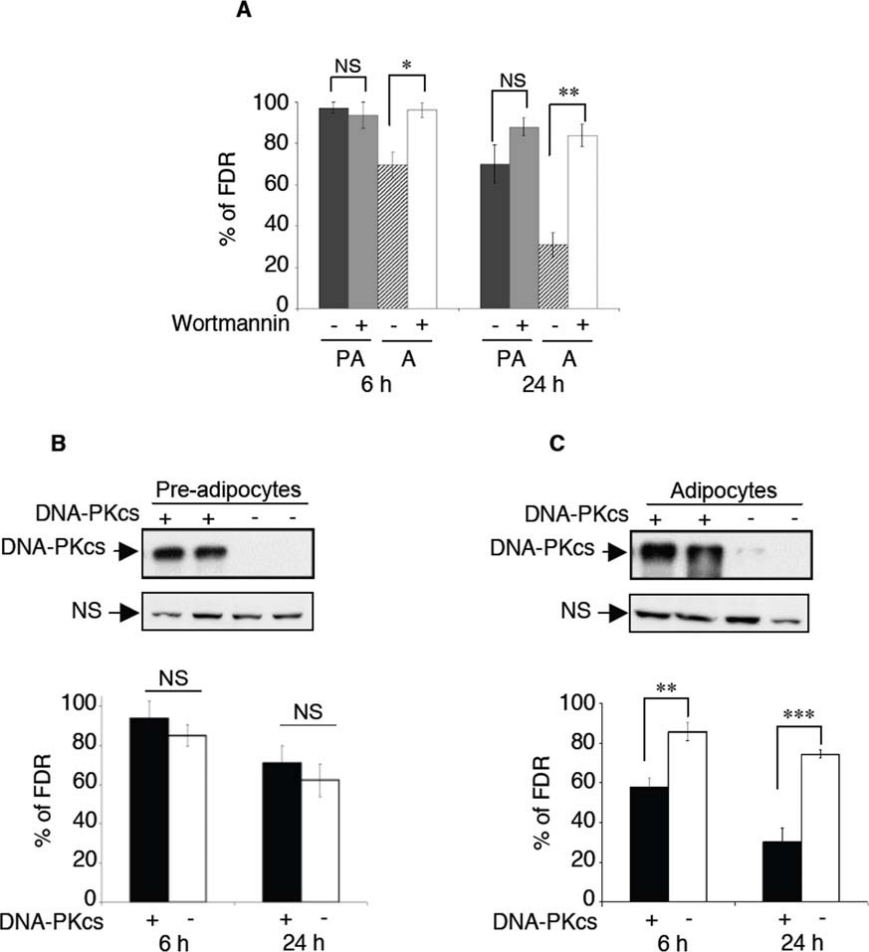
The increased ability of adipocyte to repair DSBs is strictly dependant on the classical NHEJ pathway. (A) The kinetic of DSBs repair after 1 h exposure to CLγ1 (500 pM), in presence or not of 50 µM of Wortmaninn, for pre-adipocyte and adipocyte cells, was analyzed by PFGE. Mean of 3 independent experiments±SEM. *Statistically significant by Student's *t*-test, *P*<0.05 relative to control without wortmannin; ** statistically significant by Student's *t*-test, *P*<0.01 relative to control without wortmannin. (B) DNA-PKcs expression in pre-adipocytes 3T3F442A clones stably expressing shRNA sequences directed against either DNA-PKcs mRNA or shRNA control (upper panel) and kinetic of DSBs repair performed by PFGE after treatment with 500 pM of CLγ1 (1 h) and post incubation in drug-free medium for 6 or 24 h (lower panel)(C) Similar experiments were performed with the two same clones after differentiation into adipogenic medium. Mean of 3 independent experiments±SEM. **Statistically significant by Student's *t*-test, *P*<0.01 relative to control; *** statistically significant by Student's *t*-test, *P*<0.001 relative to control. The expression of DNA-PKcs was evaluated by Western blots experiments.

We next examined if the higher rate of DSBs DNA repair observed in differentiated adipocytes was related to the increase of DNA-PK activity. Different sets of experiments were performed. First, we investigated whether or not the increase in DNA-PKcs expression was associated with an increase in DNA DSB repair activity during the first day following the commitment into differentiation. An increase in DSBs repair activity was observed in insulin treated, 24 h post-confluent 3T3F442A cells as compared to 80% confluent cells at the beginning of the treatment (residual DNA breaks 24 h after exposure to IR: 24±3.8 versus 66±8.6% respectively, p<0.05, cells exposed for 1 h to 500 pM CLγ1). Thus, increase of DNA DSBs repair activity was an early event during adipogenesis occurring within the same time period than the increase in DNA-PKcs expression. Secondly, DNA DSBs repair activity was measured in the presence or not of the fungal metabolite wortmannin that has been shown to inhibit the DNA DSBs repair in a DNA-PK dependent manner in murine and human cells [40]–[42]. We determined in preliminary experiments that wortmannin treatment lead to an inhibition of DNA-PK activity of more than 80% in the two cell types (data not shown). As shown in Fig. 4A, wortmannin (50 µM) was able to almost completely inhibit the DNA DSBs repair activity in differentiated adipocytes whereas a limited and not significant effect was observed on the repair kinetics of preadipocytes. Finally, to confirm the specific implication of DNA-PK in the observed effects, we use long term RNA interference to obtain clones of 3T3F442A cells that do no longer expressed DNA-PKcs. As shown in Fig. 4B (upper panel), we obtained two independent clones of pre-adipocytes exhibiting a very strong down-regulation of DNA-PKcs expression and we assessed that this down-regulation was further maintained in differentiated adipocytes (Fig. 4C, upper panel). There is accumulating evidence that DNA breaks accumulate during some differentiation processes such as maturation of spermatides, embryo development, and differentiation of myotubes, epidermal cells lymphocytes and neutrophils [43] and as such potentially participate to the regulation of differentiation programs. Accordingly, we evaluate adipocyte differentiation in the DNA-PK deficient, as compared to DNA-PK proficient, F442A sub-clones. No differences in the levels and time occurrence of the expression of early (aP2) or late (HSL) differentiation related genes (determined by qPCR) were observed (data not shown) showing that the up-regulation of DNA-PK is not a required event to the differentiation process. Using these two clones, we clearly demonstrated that the down-regulation of DNA-PKcs expression specifically down-regulates DNA DSBs repair activity in adipocytes (see Fig. 4C, lower panel) but not pre-adipocytes (Fig. 4B, lower panel). Taken together, these results showed using three different approaches that a DNA-PK dependent DSBs repair activity is specifically acquired during adipogenesis.

### Positive regulation of DNA-PKcs expression also occurs during human adipocyte differentiation

**Figure 5:**
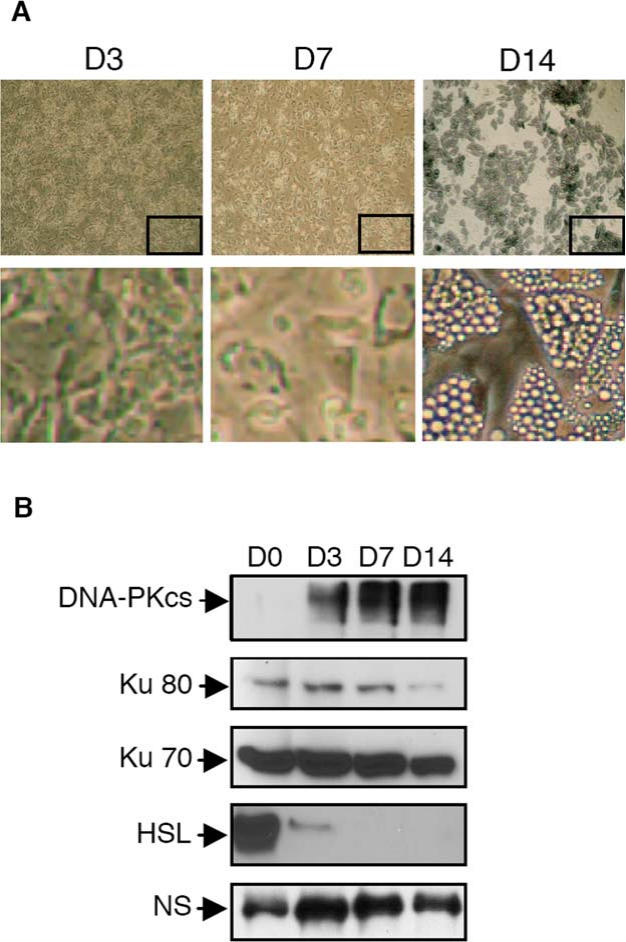
Expression of DNA-PKcs is enhanced during adipogenesis of human pre-adipocytes isolated from sub-cutaneous adipose tissue. Cells isolated from the stromal vascular fraction of collagenase digested subcutaneous tissue were grown for the indicated times in adipogenic medium as described in the Material and Methods section. (A) Photographs of the cultured cells and (B) Western blots experiments using indicated antibodies. NS, non specific band that show equal loading between the different samples.

Although 3T3F442A cells are valuable experimental model, they do have distinct attributes compared with human cells in primary culture, beyond the obvious species differences. For example, 3T3F442A cells are of embryonic origin and such features could potentially influence DNA damage signaling and repair. Thus, we investigated the regulation of DNA-PKcs expression of human preadipocytes purified from abdominal subcutaneous adipose tissue. Using a recently described approach [21] we were able to differentiate these primary cells reproducibly and efficiently with about 75% of preadipocytes converted to the adipocyte phenotype (see Fig. 5A). Using cell extracts prepared at different time point during adipose conversion, we show that in all the samples tested (n = 3 independent donors, see Fig. 5B for a representative Western blot experiment), the level of DNA-PKcs expression increase during the early commitment in adipocyte differentiation (D3). The level of DNA-PKcs expression was further maintained in the differentiated adipocyte (D7 and D14). Accordingly, DNA-PK kinase activity increases during adipogenesis by 4 and 3- fold in D3 and D7 cells respectively as compared to D0 (mean of value obtained from two independents donors).

## Discussion

In this paper, we demonstrate that adipocyte differentiation, as a representative of a differentiation program of long-lived post-mitotic cells, is associated with an early up-regulation of DNA DSBs repair activity and over-expression of one of the key factors of NHEJ, DNA-PKcs. Using pharmacological inhibition of DNA-PK activity and DNA-PK deficient adipocytes (obtained from long term RNAi directed against DNA-PKcs mRNAs), a functional link between this two events was established. First, our data show that the expression of the DNA-PKcs is the limiting component of the holoenzyme activity since a 2 to 3-fold over-expression (without modification of the level of its regulatory sub-unit Ku), as observed in differentiated adipocytes as compared to pre-adipocytes (either exponentially growing or at confluence) lead to a clear increase in DNA-PK activity and a related increase in DNA DSBs repair (see Fig. 3 and 4). These results are in agreement with previously published data since several lines of evidence have indicated that an over-expression of DNA-PKcs alone may provide important protection from genotoxic stress related to its involvement in DNA repair. For example, we have previously demonstrated that an increase in DNA-PKcs expression and activity, and a related increase in DNA DSBs repair, was observed in radioresistant HeLa cells over-expressing the 24 kDa FGF2 isoform [44]. Furthermore, exposure of the cells to nitric oxide results in a 2 to 4-fold increase in DNA-PKcs expression and the NO-generating cells have a corresponding increase in DNA DSBs activity [45]. In the two studies, the level of expression of the Ku heterodimer was unaffected [44], [45]. Limited data are available concerning the molecular mechanisms that might contribute to the up-regulation of DNA-PKcs expression. In the previously cited reports where these mechanisms have been explored, an up-regulation of DNA-PK gene transcription was found [44], [45] that was related to the binding of activated Sp1 to the DNA-PKcs promoter in the NO-dependent regulation pathway described [45]. Since *in vitro* adipogenic differentiation increases the expression levels of the inductible NO synthase (iNOS) [46], experiments were performed to test this hypothesis in our model. As shown in, no difference in the levels of DNA-PKcs mRNA was observed during both human and murine adipogenic differentiation, dismissing this hypothesis. In addition, we further assess that the observed increase in DNA-PKcs level was not affected when cells were treated with the NOS inhibitor L-NIO (data not shown). The absence of transcriptionnal regulation of DNA-PKcs suggests that adipogenic differentiation regulates either DNA-PKcs protein biosynthesis or degradation. To demonstrate whether this entirely new mechanism of regulation of DNA-PKcs expression occurs at traductional or post-traductional levels, we were confronted to the lack of antibodies able to reproducibly and quantitatively immunoprecipitate DNA-PKcs in mouse cells. Experiments already performed in our laboratory have shown that like its DNA-binding sub-unit Ku [47], the half-life of DNA-PKcs in human cells is greater than five days, demonstrating that a further increase in protein stability is unlikely to account for the observed increase in its steady state level (data not shown). Finally, it is interesting to note that rodents have up to 50-fold lower DNA-PK activity relative to human [38]. However, up-regulation of DNA-PKcs was also observed during the differentiation of human primary adipocyte clearly highlighting the fact that this event was not restricted to the relative deficiency observed in rodent cells.

It has been previously described that the NHEJ activity is predominantly used during the G0/G1 phase of the cell cycle whereas HR is down regulated in G1 and up-regulated during S/G2 phases [11]. These forms of regulation ensure that HR is maximal when appropriate homologous templates (especially sister chromatids) are available for repair and that NHEJ is maximal when such templates are not available. Differentiated adipocytes permanently exit the cell cycle in the course of acquiring functional specialization and are in G0/G1 in a so-called post-mitotic state. However, both previously published data and our present report suggest that the observed up-regulation in DNA-PKcs expression during adipogenesis is not a cell-cycle-related event. First, it is important to note that the observed increase NHEJ in G0/G1 cells is not associated with an increase in DNA-PKcs expression [48]. Furthermore, it has also been demonstrated using osteosarcoma human cell line that quiescent cells (as defined by cells grown to confluence for several days) have 5-fold less DNA-PK activity than proliferating cells [49]. Finally, the time-course of occurrence of DNA-PK over-expression and the related increase in DNA DSBs repair activity also argue against a cell-cycle related event. In fact, DNA-PKcs over-expression occurs early during the differentiation process and temporally correspond to a stage where confluent pre-adipocytes reenter the cell cycle to undergo mitotic clonal expansion [50] before the exit from the cell cycle.

Our study, in addition to the current literature, shed a new light on the link that exists between differentiation and DNA repair. Data obtained to date show that DNA DSBs repair is down regulated in certain differentiated cells such as hematopoietic cells [51], [52] or jejunum epithelium [51]. These specialized differentiated cells that are under constant turnover without a triggering event might request less efficient protecting pathways in response to DNA damage. On opposition, stem cells of these tissues, such as hematopoietic stem cells (HSC) of the more primitive phenotype, are proficient in DSB repair [53]. In addition, recent evidences have shown that NHEJ repair activity, among others DNA repair pathways, is essential for stem cell maintenance, and a decline in DNA repair function with age is observed [54]. Our results demonstrate that, in opposition to the general view that DNA DSBs repair is decreased during differentiation, an up-regulation of this process might be observed in post-mitotic long lived cells. In comparison to other cells, pre-adipocytes can be considered as relatively deficient in DNA DSBs repair activity as exemplified by the slow repair kinetic observed with significant residual DNA breaks at 24 h (see Fig. 1C). In fact, the classical DNA-PK dependent NHEJ pathway is poorly operational in these cells and, as demonstrated using two different approaches (pharmacological inhibition of DNA-PK activity and down-regulation of DNA-PKcs expression), we show that a DNA-PK dependent DSBs repair activity is specifically acquired during adipogenesis (see Fig. 4A and 4B). DNA DSBs repair in pre-adipocytes might rely on HR but also on the recently described alternative end-joining pathway, called non-classical NHEJ pathway or B-NHEJ (B standing for back-up) [24] that involves a different ligase, ligase III in complex with XRCC1 [24], [55] and probably the PARP protein [55], [56]. However, preliminary experiments performed in our laboratory show that expression of these proteins are not regulated during both murine and human adipogenesis (data not show). Although limited experimental data exists, the fact that differentiated long-lived cells acquired active mechanisms that hampers DNA DSBs effects as compared to less differentiated precursors have been previously described [57], [58]. For example, a recent report by Novak et al [58] show that the kinetics of γH2AX foci disappearance after irradiation of the developing mouse brain is faster in neurons than in neural precursors. An active process of protection against DNA DSBs has been also observed in differentiated myotubes that counteract the risk of DNA damage consequences by a selective blockade of DNA damage signaling downstream of ATM [57], a process that is not observed in the less differentiated precursors, the myoblasts. In conclusion, we have demonstrated for the first time an up-regulation of DNA DSBs repair activity during a differentiation process, that clearly underline the need for some tissues to ensure long-life maintenance upon stress conditions. It may be rewarding to examine whether this or analogous mechanisms guard against DNA-damage induced cell death in other differentiated tissue as well
